# Is the Recurrence of Fibroma of the Tendon Sheath Underestimated? An Instructive Case Report and a Review of the Literature

**DOI:** 10.1155/2020/5357329

**Published:** 2020-01-28

**Authors:** E. Lüdke, G. Kohut, H. C. Bäcker, M. Maniglio

**Affiliations:** ^1^Department of Orthopaedic Surgery, Hôpital Neuchâtelois, Switzerland; ^2^Department of Orthopaedic Surgery, HFR Hôpital Cantonal de Fribourg et Clinique Générale St-Anne, Switzerland; ^3^Department of Orthopaedic Surgery, Charité University Hospital Berlin, Germany

## Abstract

We report a case of a 21-year-old healthy woman with a history of a painful growing mass in the palm of the right hand, with a trigger finger phenomenon. The mass was surgically entirely excised, and the histological findings of the tumor were those of a fibroma of the tendon sheath (FTS) starting from the flexor tendons. Although the initial outcome was good, the patient experienced the same symptoms at the same location 4.5 years later. The MRI demonstrated a 50 × 10 × 5 mm mass of low intensity on T1-weighted images and high intensity on T2-weighted images and gadolinium enhancement. A second complete excision of the tumor was performed by the same senior surgeon, and the histology confirmed the recurrence of the FTS. We also reviewed the scientific literature about FTS in the hand. Most recent studies show a low rate or no recurrence at all. We hypothesize that a lot of recurrences are missed because of a short follow-up and that the recurrence rate may be higher than thought.

## 1. Introduction

Fibroma of the tendon sheath (FTS) is a rare and benign slow-growing soft tissue tumor that has a predilection for the palmar aspect of the wrist and the hand, mostly in male adults [1–4]. It was initially described by Buxton in 1923 [5] and further defined by Geschickter and Copeland in 1949 [6]. The etiology still remains unclear, and the notion of previous trauma is absent in most patients. Symptoms are an insidiously growing mass with tenderness or pain. Trigger finger or carpal tunnel syndrome (CTS) may also be present [7]. The first step in the diagnosis is the MRI to assess the exact size and limits of the mass. It usually shows a lobulated mass, with low intensity on T1 signal, and low to intermediate intensity, or sometimes higher intensity on T2 signal in the presence of a myxoid component [8]. The treatment consists of a surgical marginal excision. A large study over a 15-year period with 208 soft tissue hand tumors showed 3% of histologically diagnosed FTS [9]. Although a pathological study from 1979 shows a recurrence rate of up to 24% [1], the most modern literature shows few or no recurrence rate at all [3, 7, 8, 10]. This difference might be explained by improved surgical techniques and use of the tourniquet and loupes by some authors [3, 9], but we found that the recent literature shows a short follow-up period or no follow-up description at all [7, 8, 10].

In this study, we want to present a case of recurrence of FTS, 4.5 years after surgery, and to show that recurrence rate is probably underestimated because of short-term follow-up periods in most studies.

## 2. Material and Method

A 21-year-old, healthy, right-handed Caucasian woman, working as a nurse, presented with a history of limited 4th finger extension, pain in the right palm, and a trigger phenomenon in November 2012. She consulted her family doctor in December 2012 because of worsening symptoms, particularly the trigger phenomenon and the pain. She was referred to a hand surgeon. The physical examination demonstrated a sizzling noise while moving. A strong tenosynovitis was suspected, and the patient underwent an open trigger finger release and synovectomy in February 2013. A senior hand surgeon performed the surgery.

Intraoperatively, a plurilobated, grossly firm, and white-colored tumor was identified in Zone 3 of the flexor tendons of the 4th finger. The mass was 10 × 7 × 4 mm, adherent to the deep flexor tendon and peduncled to the radial side of it. The tumor was carefully dissected from the flexor tendons and the neurovascular structures and excised in toto.

The external surface of the mass was smooth and glistening without any visible abnormalities.

The lesion was histologically well circumscribed and contained proliferating synovial cells in a collagenous background. There was no mitotic atypia. The histological diagnosis was FTS, and the analysis showed that the excision was complete.

Immediately after the surgery, the 4th finger could reach complete passive motion. The pain level reduced gradually, and three months after the surgery, the patient could achieve full active range of motion of the 4th finger with no tenderness. She had the last medical control 2 months after the surgery and stopped the ergotherapy 3 months postoperatively.

She returned to her job 3 weeks after the surgery and felt only a lack of force in the ring finger, with a complete recovery after 4 months. No further problems or symptoms until May 2017 were reported, when she started to present with identical symptoms, a new rising tumefaction in the scar area in the last 3 weeks. She complained again of a reduced finger function, and the physical examination showed an active extension of the 4th finger of metacarpophalangeal joint: 30°; proximal interphalangeal joint: 60°. The passive motion was not limited, and some pain in the palm of the right hand while moving was present.

**Figure 1:**
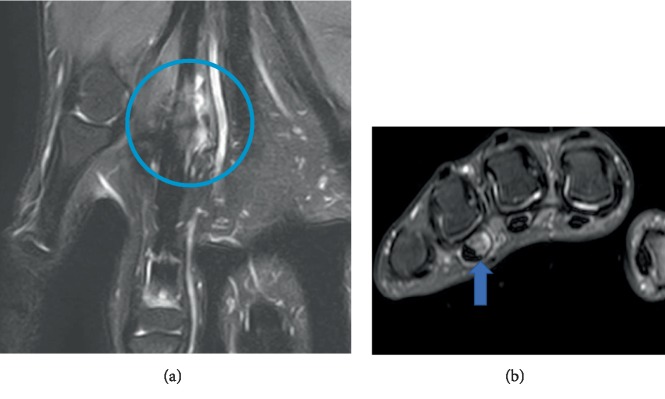
(a) MRI T2 sequence: coronal view of the hand. (b) MRI T2 sequence: axial view of the hand.

A magnetic resonance imaging (MRI) was performed and confirmed the diagnosis of a recurrent FTS, revealing a 50 × 10 × 5 mm mass of low intensity on T1-weighted images and high intensity compared with normal muscle on T2-weighted images and gadolinium enhancement (Figures 1(a) and 1(b)).

**Figure 2:**
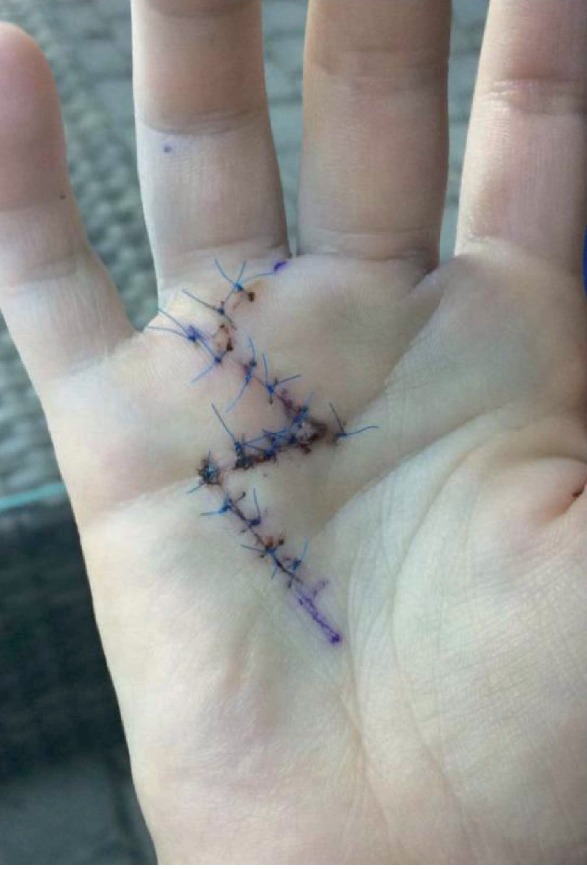
Second surgery: Brunner incision.

**Figure 3:**
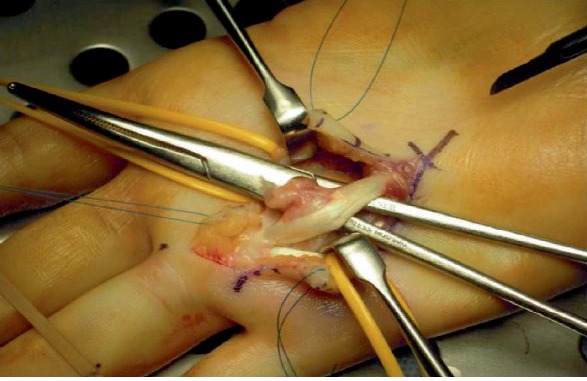
Second surgery: tumor before removal.

**Figure 4:**
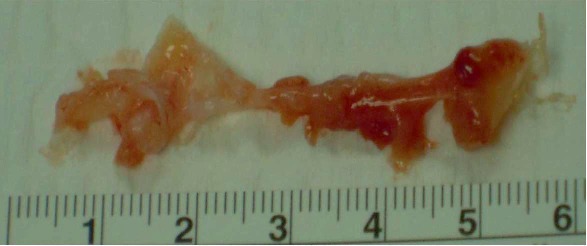
Second surgery: excised tumor.

A second surgery was performed in June 2017 by the same senior hand surgeon. The surgical approach was done through the scar of the 1st surgery, extended with a Brunner incision (Figure 2). The cicatrized palmar aponeurosis upon the 4th flexor tendons was resected. The tumor was adherent to the flexor digitorum profundus of the 4th finger, starting between its fibers. The aspect of the tumor was similar to the initial one: firm and white-colored, with a smooth and glistening external surface (Figures 3 and 4). The tumor was excised in toto with a marginal resection, including the epitendineum, the distal part of the origin of the 4th lumbricularis muscle and some adherent fibers of the deep flexor tendon. Distally, the tendon sheath was resected and the pulley A1 was excised. All these visual findings do suggest a source of recurrence, crystalizing the theory that there was probably residual tumor within the tendon fibers or the epitendineum after the first surgery.

**Figure 5:**
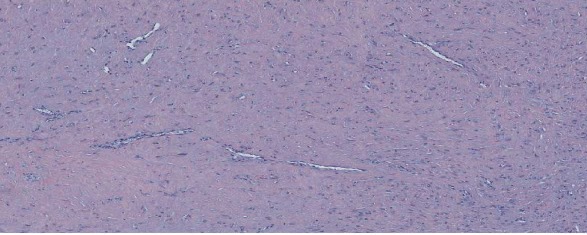
Histology of FTS.

Histologically, the lesion was overlayable with the first lesion, containing fibroblastic spindle cells without cytonuclear atypia in a collagenous background, some hemosiderin deposits, and small intralesional vessels (Figure 5). No inflammatory reaction or signs of malignity were found.

The histological diagnosis was clearly a FTS, without necessity for immunohistochemistry.

It showed a local recurrence, 4.5 years after the first complete surgical excision of the tumor.

The healing time lasted longer than after the first surgery, and the patient complained of a loss of sensitivity in the palmar aspect of the third phalanx, with spontaneous resolution after 3 months. An ergotherapy treatment was started postoperatively during 2 months. The grip strength (Jamar test) was diminished in the right hand with 16 kgs versus 26 kgs in the left hand after the surgery. At the end of the ergotherapy treatment, the Jamar showed an almost completely recovered strength of 24 kgs.

Two years after the second surgery, the patient has no more pain and her finger recovered complete active range of motion. She only complains of a subjective impression of diminished strength in the finger and estimates the force at about 70-80% compared to the left ring finger.

## 3. Discussion

FTS is a rare and benign slow-growing soft tissue tumor initially described by Buxton in 1923 [5]. It may occur throughout the body, but in a large series of 138 cases, in 1979, Chung and Enzinger reported [1] that 98% of the tumors originate in the extremities and 81.8% occur in the wrist, hand, and fingers. They also noted that the tumor was likely located on the palmar surface of the hand, mostly in the right hand. Men were predominantly affected, with an age of incidence between the third and fifth decades of life. A recurrence rate of 24% was observed after surgical excision. The interval between the initial surgery and the recurrence varied from a range of 1 month to 5 years. We objectified no particular factor that could be associated with the recurrence in the literature, such as trauma, particular jobs, or dominant-nondominant hand.

Millon et al. mainly focused on soft tissue hand tumors in a large study over a 15-year period. Three percent of the 208 lesions were histologically diagnosed as FTS [9]. Like Al-Qattan [3], they reported no recurrence at all and imputed their results to an aggressive resection of lesions. Therefore, they removed the neurovascular structures when they were adhering to the tumors, accepting a possible decreased function of the member postsurgically.

Actually, the etiology of FTS remains unclear, and in most studies, the notion of previous trauma is absent. Only 3 patients out of 18 (17%) in the study of Hashimoto et al. [11] and 2 patients out of 32 (6%) in the study of Pulitzer et al. [2] are known to have had a past trauma in the zone of the tumor.

While Al-Qattan reported the FTS in a series of 23 slow-growing and painless mass of the hand [3], some authors reported it as an unusual cause of carpal tunnel syndrome in 2011 [7, 10, 12].

Although MRI is usually used as the first step in the diagnosis, a definite diagnosis cannot be made on the basis of only the MRI characteristics. However, we think that MRI is not (yet) accurate enough to see if the mass is intratendinous or only in contact with the tendon. This has to be evaluated intraoperatively and should lead to a more aggressive resection in case of a tendon involvement of the tumor.

Histologically, the FTS presentation is quite similar to common giant cell tumors of the tendon sheath but is distinguished by the lack of giant cells, synovial cells, and foamy histiocytes [8]. It appears as multilobular spindle or stellate-shaped fibroblasts with elongated nuclei within a dense collagenous stroma, without necrosis or inflammatory cells [1, 3, 4, 8, 9]. Differential diagnosis has to include fibromatosis, nodular fasciitis, neurofibroma, leiomyoma, fibrous histiocytoma, scar tissue, and giant cell tumor [1, 4, 9]. The giant cell tumor is the most common benign tumor of the tendon sheath and may be easily confused with FTS. Although its clinical presentation and its microscopic appearance are very similar to FTS, they can be differentiated microscopically. Giant cell tumor contains proliferating synovial cells, foamy histiocytes, and giant cells, all absent in FTS [9].

Our case shows a late recurrence of FTS with the reappearance of a trigger finger and pain, more than 4 years after the complete excision of the mass. Most of the recent studies would have missed this late recurrence. Like what Millon et al. and Al-Qattan reported [3, 9], a more aggressive excision, with the resection of the neurovascular structures that are adherent to the tumor, is the first line of a successful treatment and it can explain the diminution of the recurrence rate.

In our opinion, as Smith et al. suggested [4], Chung and Enzinger reported a recurrence rate of 24% with a long follow-up period in their large series in 1979 [1]. However, since then, most studies show very low or zero recurrence rates (Table 1) which are not only explained by the use of intraoperative magnification and tourniquet but possibly by inadequate follow-up. As showed in Table 1, most recurrences appear in the interval of 3 months to 5 years postoperatively, as in our case. Thus, we do think that most FTS late recurrence are currently missed.

## 4. Conclusion

FTS is a very rare cause of trigger finger, pain, or even CTS. The treatment consists of a surgical marginal excision. Although the recurrence rate could be diminished with improved and sometimes more aggressive surgical techniques, it is probably underestimated because of the short-term follow-up of recent studies. We suggest that the follow-up should last for at least 3 years. We also suggest that the future literature should be more attentive in recognizing factors associated with the recurrence.

## Figures and Tables

**Table 1:** Summary of the scientific articles about FTS with treatment, mean FU, and recurrence rate.

Authors	Numbers	Treatment	Mean FU	Recurrence rate	Recurrence time
Millon et al. [9]	7	Surgical marginal excision	8 years	None	—

Hashimoto et al. [11]	18	Surgical excision (no precision)	6.4 years for 15 patients	3 patients: 1 recurrence (20%)	3 months, 11 months, 2 years

Chung and Enzinger [1]	138	Surgical excision (no precision)	4.5 years for 54 patients	10 patients: 1 recurrence (18%) 3 patients: 2 recurrences (6%)	13.1 months to 5 years

Al-Qattan [3]	20	Surgical excision under local anesthesia	3.2 years	None	—

Pulitzer et al. [2]	32	Surgical excision (no precision)	?	1 (3%)	?

Smith et al. [4]	9	No explanation	?	1 (11%)	5 months
